# A safe and convenient pseudovirus-based inhibition assay to detect neutralizing antibodies and screen for viral entry inhibitors against the novel human coronavirus MERS-CoV

**DOI:** 10.1186/1743-422X-10-266

**Published:** 2013-08-26

**Authors:** Guangyu Zhao, Lanying Du, Cuiqing Ma, Ye Li, Lin Li, Vincent KM Poon, Lili Wang, Fei Yu, Bo-Jian Zheng, Shibo Jiang, Yusen Zhou

**Affiliations:** 1 State Key Laboratory of Pathogen and Biosecurity, Beijing Institute of Microbiology and Epidemiology, Beijing, China; 2 Lindsley F. Kimball Research Institute, New York Blood Center, New York, NY, USA; 3 Department of Microbiology, the University of Hong Kong, Hong Kong, China; 4 Key Laboratory of Medical Molecular Virology of Ministries of Education and Health, Shanghai Medical College and Institute of Medical Microbiology, Fudan University, Shanghai, China

**Keywords:** Novel human coronavirus, MERS-CoV, Spike protein, Pseudovirus, Neutralizing antibodies, Antiviral therapeutics

## Abstract

**Background:**

Evidence points to the emergence of a novel human coronavirus, Middle East respiratory syndrome coronavirus (MERS-CoV), which causes a severe acute respiratory syndrome (SARS)-like disease. In response, the development of effective vaccines and therapeutics remains a clinical priority. To accomplish this, it is necessary to evaluate neutralizing antibodies and screen for MERS-CoV entry inhibitors.

**Methods:**

In this study, we produced a pseudovirus bearing the full-length spike (S) protein of MERS-CoV in the Env-defective, luciferase-expressing HIV-1 backbone. We then established a pseudovirus-based inhibition assay to detect neutralizing antibodies and anti-MERS-CoV entry inhibitors.

**Results:**

Our results demonstrated that the generated MERS-CoV pseudovirus allows for single-cycle infection of a variety of cells expressing dipeptidyl peptidase-4 (DPP4), the confirmed receptor for MERS-CoV. Consistent with the results from a live MERS-CoV-based inhibition assay, the antisera of mice vaccinated with a recombinant protein containing receptor-binding domain (RBD, residues 377–662) of MERS-CoV S fused with Fc of human IgG exhibited neutralizing antibody response against infection of MERS-CoV pseudovirus. Furthermore, one small molecule HIV entry inhibitor targeting gp41 (ADS-J1) and the 3-hydroxyphthalic anhydride-modified human serum albumin (HP-HSA) could significantly inhibit MERS-CoV pseudovirus infection.

**Conclusion:**

Taken together, the established MERS-CoV inhibition assay is a safe and convenient pseudovirus-based alternative to BSL-3 live-virus restrictions and can be used to rapidly screen MERS-CoV entry inhibitors, as well as evaluate vaccine-induced neutralizing antibodies against the highly pathogenic MERS-CoV.

## Background

In April, 2012, a severe acute respiratory syndrome (SARS)-like disease emerged in Saudi Arabia [1-3]. As of August 13, 2013, the World Health Organization (WHO) had received reports of 94 cases of infection caused by this novel human coronavirus, Middle East respiratory syndrome coronavirus (MERS-CoV, previous name hCoV-EMC), including 47 deaths, from several countries, including Saudi Arabia, Qatar, Jordan, the United Arab Emirates, the United Kingdom, France, Germany, Italy, and Tunisia (http://www.who.int/csr/disease/coronavirus_infections/update_20130813/en/). Eight MERS-CoV clusters have been reported, suggesting person-to-person transmission of the disease [4]. However, the transmissibility of MERS-CoV appears to be less efficient than that of SARS-coronavirus (SARS-CoV), the first new infectious disease identified in the 21st century with an approximate mortality rate of 10% [5].

Genetically, MERS-CoV is closely related to SARS-CoV [1,6]. Clinically, severe respiratory illness with renal failure caused by MERS-CoV infection is very similar to the symptomology related to SARS [2]. Therefore, the outbreak of MERS-CoV infection has raised serious concerns of a potential global pandemic on the order of SARS in 2003. Accordingly, pursuant to the development of effective vaccines and antiviral agents, the first step requires identification of the neutralizing and inhibitory activities of such anti-MERS-CoV vaccines and therapeutics.

It is now well known that SARS-CoV gains cellular entry through its receptor, angiotensin converting enzyme 2 (ACE2) [7], whereas MERS-CoV utilizes dipeptidyl peptidase-4 (DPP4, also known as CD26) as its entry receptor [8]. Similar to SARS-CoV, the spike (S) protein of MERS-CoV also plays important roles in receptor binding and viral entry [5,9]. As the major protein causing virus infection, S protein is an ideal target for both vaccines and MERS-CoV entry inhibitors.

In this study, we produced a pseudovirus bearing the full-length S protein of MERS-CoV in the Env-defective, luciferase-expressing HIV-1 backbone. We then established a pseudovirus-based inhibition assay for the detection of neutralizing antibodies and anti-MERS-CoV entry inhibitors. This method was proven to be safe, convenient, reliable and effective for the rapid detection of neutralizing antibodies and viral entry inhibitors against MERS-CoV.

## Results

### Generated MERS-CoV pseudovirus was able to infect a variety of cell types from human and non-human hosts

**Figure 1:**
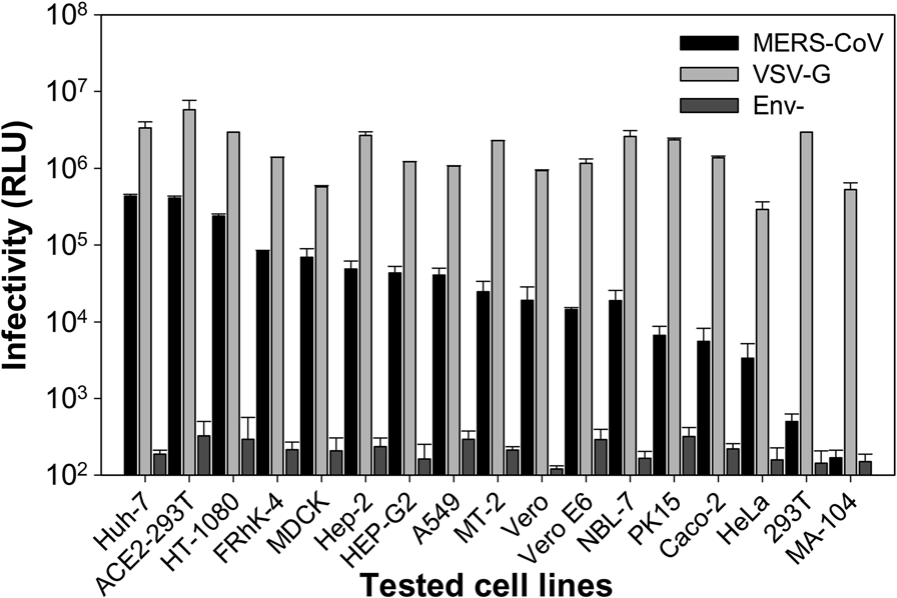
**Detection of MERS-CoV pseudovirus infectivity.** Cell tropism of MERS-CoV pseudovirus in a variety of target cells from human and non-human hosts. VSV-G and Env- pseudoviruses were used as positive and negative controls, respectively. The data are expressed as mean relative luciferase units (RLU) ± standard deviation (SD) of 4 parallel wells in 96-well culture plates. The experiment was repeated three times, and similar results were obtained.

We first detected the ability of the generated MERS-CoV pseudovirus to infect cells from various tissues and different hosts, including human cell lines Huh-7, HT-1080, Hep-2, HEP-G2, A549, MT-2, Caco-2, HeLa and 293T, as well as those from mink (NBL-7), pig (PK15), canine (MDCK), and monkey (FRhK-4, Vero, Vero E6 and MA-104) (Table 1). Pseudovirus expressing VSV-G was included as the positive control. The target cells were respectively infected with MERS-CoV pseudovirus normalized for equal HIV-1 p24 content, and luciferase activity was measured at 72 h after infection. As shown in Figure 1, almost all tested human cells and a variety of animal cells could be infected by the produced pseudovirus. Particularly, MERS-CoV pseudovirus infected HT-1080 and SARS receptor-expressing ACE2-293T cells, maintaining a high infective ability in identified MERS-CoV receptor DPP4-expressing Huh-7 cells. In addition, human cell types, such as Hep-2, HEP-G2, A549 and MT-2, and animal cell types, including FRhK-4, MDCK, Vero, Vero E6 and NBL-7, were infected by MERS-CoV pseudovirus to a significantly higher degree than other cell types, such as PK15, Caco-2 and HeLa. However, no high infectivity was observed in MA-104. Our results also demonstrated that VSV-G positive control could highly infect almost all tested cell types, possibly due to the broad host range of VSV-G in the capability of infecting multiple tissues in various hosts, while Env- pseudovirus negative control was unable to infect tested cell lines [10] (Figure 1).

**Table 1:** Cell lines used for detection of MERS-CoV pseudovirus infectivity and receptor DPP4 expression

**Cell lines**	**Origin**	**Provider**	**Note**
Huh-7	Human liver	Dr. Charles M. Rice at Rockefeller University	
HEP-G2	Human liver	ATCC	
HT-1080	Human fibrosarcoma	ATCC	
MT-2	Human lymphocyte	NIH AIDS Reagent Program	
Hep-2	Human respiratory tract	ATCC	
Caco-2	Human intestinal tract	ATCC	
HeLa	Human genitourinary tract	ATCC	
293T	Human kidney	ATCC	
ACE2-293T	293T-derived cells	Laboratory stock	Express SARS-CoV receptor ACE2
A549	Human lung	ATCC	
NBL-7	Mink lung	ATCC	Mv1Lu
PK15	Pig kidney	ATCC	
MDCK	Canine kidney	ATCC	
FRhK-4	Fetal rhesus monkey kidney	ATCC	
Vero	African green monkey kidney	ATCC	
Vero E6	African green monkey kidney	ATCC	Vero C1008
MA-104	African green monkey kidney	ATCC	

**Figure 2:**
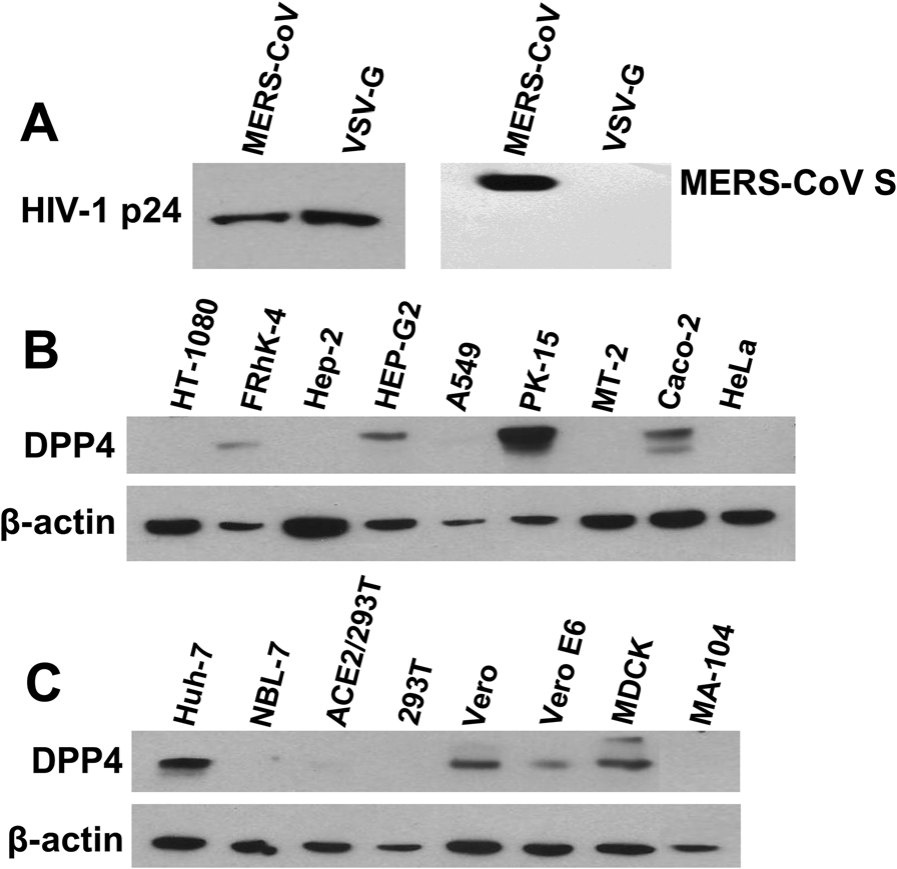
**Detection of MERS-CoV S and HIV-1 p24 protein expression in the packaged MERS-CoV pseudovirus and DPP4 protein expression in target cells by Western blot. (A)** Detection of p24 and MERS-CoV S in pseudotyped MERS-CoV. Anti-p24 (1:50) and MERS-CoV S-specific polyclonal antibodies (1:200) were used for the test. **(B**-**C)** Detection of DPP4 expression in different cell lines. Goat anti-DPP4 (1 μg/ml) was used for the test, and anti-β-actin monoclonal antibodies (1:5,000, Sigma) were applied as the internal control.

Next, we performed Western blot to identify the incorporation of MERS-CoV S in the packaged MERS-CoV pseudovirus. As shown in Figure 2A, clear bands corresponding to the HIV-1 p24 and MERS-CoV S protein were identified in the generated pseudovirus of MERS-CoV by using antibodies against HIV-1 p24 and MERS-CoV S protein, respectively, while there was only p24, but no MERS-CoV S antigen, was detected in the VSV-G pseudovirus containing p24. These data suggest that specific S protein of MERS-CoV was effectively incorporated into the packaged HIV-1 particle, generating MERS-CoV pseudovirus. Western blot was also carried out to detect the expression of DPP4 in cells susceptible to pseudotyped MERS-CoV. Among the tested cells, PK-15 exhibited the highest expression of DPP4, followed by Huh-7. While DPP4 was expressed in FRhK4, HEP-G2, Caco-2, Vero, Vero E6, and MDCK cells, a relatively low level of this protein was detected in the cells of A549 and ACE2/293T cells. Nevertheless, no DPP4 expression was shown in HT-1080, Hep-2, MT-2, HeLa, NBL-7, 293T and MA-104 (Figure 2B-C).

### MERS-CoV pseudovirus inhibition assay reliably detected the neutralizing activity of vaccinated animal sera, and the result was consistent with that of live MERS-CoV-based inhibition assay

**Figure 3:**
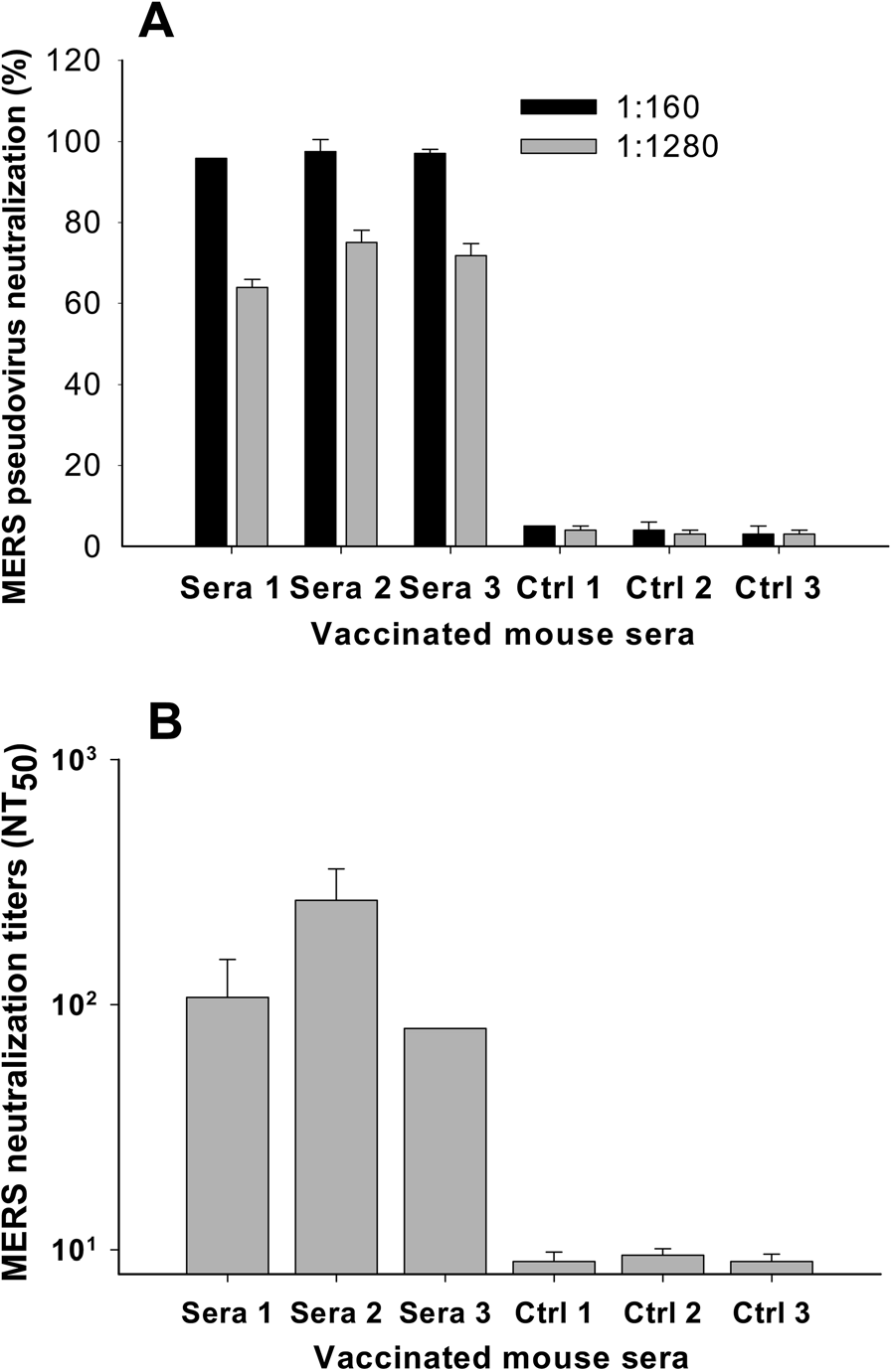
**Detection of neutralizing antibodies of vaccinated sera against MERS-CoV infection.** Mice were vaccinated with a recombinant protein expressing RBD of MERS-CoV S protein, and representative sera (Sera 1–3) collected from 10 days post-2^nd^ vaccine were used for the test. Control sera were collected from mice injected with PBS. **(A)** MERS-CoV pseudovirus-based inhibition assay in DPP4-expreesing Huh-7 cells. The data are presented as mean percentages of inhibition ± SD of duplicate wells. **(B)** MERS-CoV live virus-based inhibition assay in Vero E6 cells. The titers were determined as the highest serum dilutions that completely prevent CPE in at least 50% of the wells (NT_50_) and are expressed as mean ± SD. The experiment was repeated three times, and similar results were obtained.

We used MERS-CoV pseudovirus to establish a pseudovirus inhibition assay and evaluated the neutralizing activity in the sera of mice vaccinated with a recombinant protein, S-RBD-Fc, containing receptor-binding domain (RBD, residues 377–662) of MERS-CoV spike (S) fused with Fc of human IgG [11]. As shown in Figure 3A, three tested representative mouse sera demonstrated neutralizing activity (>96%) against MERS-CoV pseudovirus infection in the DPP4-expressing Huh-7 cells at titers of 1:160, while the control sera from mice immunized with PBS showed no neutralizing activity against tested pseudovirus. The above sera were further evaluated for neutralizing activity against MERS-CoV infection using a live MERS-CoV-based inhibition assay. The results indicated that neutralizing antibodies of these sera (test sera 1–3 and control sera 1–3) corresponded to those tested by pseudovirus inhibition assay (Figure 3B), suggesting that the established pseudovirus-based inhibition assay is sufficiently reliable to evaluate neutralizing antibodies induced by candidate vaccines against MERS-CoV.

### MERS-CoV pseudovirus inhibition assay could be used to effectively screen for MERS-CoV entry inhibitors

**Figure 4:**
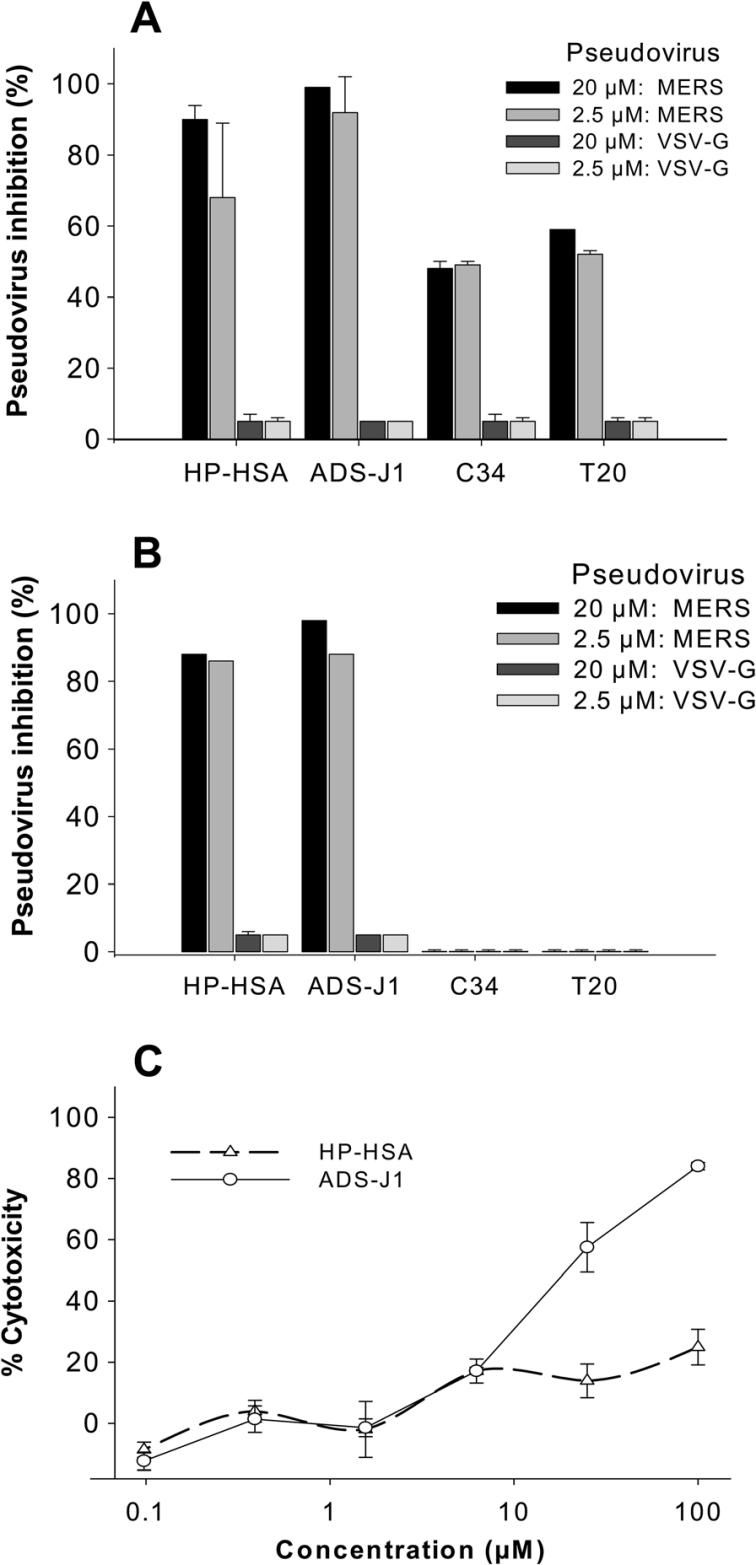
**Detection of inhibitory ability of synthetic compounds against MERS-CoV pseudovirus infection.** Compounds (HP-HSA and ADS-J1) and peptides (C34 and T20) were tested for the inhibition of MERS-CoV pseudovirus entry into target NBL-7 **(A)** and Huh-7 cells **(B)** at concentrations of 20 and 2.5 μM, respectively. VSV-G pseudotype was included as the negative control. (3) Detection of the potential cytotoxicity of the compounds to Huh-7 cells. The data are presented as mean percentages of inhibition (or cytotoxicity) ± SD of duplicate wells. The experiment was repeated three times, and similar results were obtained.

Using the established method, we next detected the inhibition activity of several HIV entry inhibitors for their inhibitory activity on MERS-CoV entry in the NBL-7 and Huh-7 cells. As shown in Figure 4A, a small molecule HIV entry inhibitor targeting gp41 (ADS-J1) [12,13] and the 3-hydroxyphthalic anhydride-modified human serum albumin (HP-HSA) targeting HIV-1 gp120 and HIV-1 receptor, CD4 [14] could inhibit >90% and >68% of MERS-CoV pseudovirus infection at the concentration of 20 and 2.5 μM, respectively, when tested in NBL-7 cells. However, the highly potent peptidic HIV entry inhibitors, C34 and T20 [15], showed moderate inhibitory activity on MERS-CoV pseudovirus infection at 2.5 and 20 μM. To further confirm our results, we detected the inhibitory ability of HP-HSA and ADS-J1 compounds as well as C34 and T20 peptides in the DPP4-expressing cell line Huh-7 [8]. Interestingly, the results were similar to those for the same compounds tested in NBL-7 cells, with around 90% of MERS-CoV pseudovirus entry inhibition at concentrations of 20 μM (Figure 4B). These two compounds, which carry net negative charges, may interact with the positively charge residues in the spike protein of the pseudotyped MERS-CoV, in a similar way as they inhibited infection of HIV [12-14]and other enveloped viruses, such as inhibition of SARS-CoV infection by ADS-J1 [16] and inhibition of human papilloma virus (HPV) infection by HP-HSA [17]. However, C34 and T20 peptides at 20 μM did not show any inhibitory activity in Huh-7 (Figure 4B), suggesting that these peptides are unable to inhibit MERS-COV pseudovirus entry into the DPP4-expressing Huh-7 cells. As expected, none of the compounds inhibited pseudotypes containing VSV-G when tested in NBL-7 and Huh-7 cell lines (Figure 4A and 4B).

We then detected the potential cytotoxicity of the identified compounds to Huh-7 cells that were used for testing their inhibitory activity on MERS-CoV pseudovirus. As shown in Figure 4C, HP-HSA had no significant cytotoxicity at the concentration as high as 100 μM. ADS-J1 exhibited some cytotoxicity to Huh-7 cells with CC_50_ (the concentration causing 50% cytotoxicity) of 26.9 μM. However, its IC_50_ (the concentration causing 50% inhibition of MERS-CoV pseudovirus infection) is 0.6 μM and its selective index (CC_50_/IC_50_) is about 45. These data indicated that the decreased infectivity of the peudotyped MERS-CoV in the presence of these compounds is not due to their cytotoxicity, but is associated with their inhibition on MERS-CoV entry. These results confirmed that the established pseudovirus inhibition assay could be used as an effective way to rapidly screen MERS-CoV entry inhibitors.

## Discussion

Angiotensin-converting enzyme 2 (ACE2) has been confirmed as the receptor of SARS-CoV [7], while MERS-CoV has been shown to bind target cells using DPP4 as its receptor [8]. In this study, we generated a pseudotyped MERS-CoV based on the viral surface S protein, and detected its infectivity in different cell types. Indeed, the generated pseudovirus was able to infect a variety of cell lines, including human and non-human cell types. Generally, its cell susceptibility was consistent with that of wildtype MERS-CoV reported previously [18], being able to maintain high infectivity in DPP4-expressing cells, such as Huh-7, MDCK, Vero, Vero E6, FRhK4, HEP-G2, and A549, and incapable of infecting HeLa and MA-104 cells with no DPP4 expression. Its infectivity to DPP4-expressing PK-15 and Caco-2 cells was relatively lower than that of wildtype virus [18]. This is possibly because that pseudotyped MERS-CoV, which contains only viral S protein with ability of single-cycle infection but lacks the capability for multiple rounds of replication, might have lower infectivity than wildtype MERS-CoV that contains all viral proteins necessary for virus replication.

It is noted that MERS-CoV pseudovirus had a relatively higher infectivity in HT-1080, Hep-2, MT-2 and NBL-7 cells with undetectable DPP4 expression, suggesting that the infectivity of MERS-CoV pseudovirus might not be completely associated with the expression level of DPP4. Similar phenomena were observed by Pohlmann and the colleagues, showing that the lentiviral pseudotype had a lower infectivity in DPP4-expressing A549 and that 293T cells from different batches maintained variant infectivities [9]. Reports have shown that HEK cells with undetectable DPP4 had relatively higher infectivity to wildtype MERS-CoV [18]. It may be possible that like SARS-CoV, which has alternative receptors (such as DC-SIGN and/or L-SIGN) in addition to ACE2 [5,19,20], MERS-CoV might also have an alternative receptor that has not been identified so far.

Notably, the peptides derived from the HIV-1 gp41 HR2 region, C34 and T20, had a moderate inhibitory activity on MERS-CoV entry into NBL-7 cells, but exhibited no inhibition on MERS-CoV entry into Huh-7 cells. These results suggest that C34 and T20 peptides may interact with the HR1 conformation of MERS-CoV S protein S2 subunit induced by the binding of S1 subunit to the not-yet-identified alternative receptor on NBL-7 cells. However, these peptides may not be able to interact with the HR1 conformation of MERS-CoV S protein S2 subunit induced by the binding of S1 subunit to the well-defined receptor DPP4 on Huh-7 cells.

Live virus-based inhibition assays have generally been used to detect neutralizing antibodies against infection of SARS-CoV or other coronaviruses [21,22]. However, to carry out this type of inhibition assay, it is necessary to utilize biosafety level 3 (BSL-3) facilities. Unfortunately, this strict condition is often inconvenient and cannot be accessed by many researchers. In contrast, our pseudovirus-based inhibition assay can be performed without the requirement of BSL-3 laboratories, considerably simplifying the detection of neutralizing antibodies, as this method does not involve live viruses and is, therefore, safe to carry out [10,23]. Moreover, we demonstrated consistency of neutralization results between the live virus-based inhibition assay and the established pseudovirus-based inhibition assay. These neutralizing results are also consistent with those obtained from the experiments on antisera of mice immunized by another RBD fragment containing residues 358–588 of MERS-CoV S protein, as reported recently [24]. The above results suggest a viable alternative for the rapid detection of neutralizing antibodies against MERS-CoV natural infection and the preclinical evaluation of candidate vaccines when BSL-3 facilities are not available. Furthermore, in the absence of BSL-3 facilities, the pseudovirus-based inhibition assay provides a convenient and reliable way to rapidly screen compound- or peptide-based MERS-CoV entry inhibitors to test neutralizing antibodies. With easy production of pseudovirus, rapid read-out and detection of inhibition, this method is particularly suitable for handling a large variety of samples in a short period of time.

## Conclusion

Our study showed that the established MERS-CoV inhibition assay is a safe and convenient pseudovirus-based alternative to BSL-3 live-virus restrictions and can be used to rapidly screen MERS-CoV entry inhibitors, as well as evaluate vaccine-induced neutralizing antibodies against the highly pathogenic MERS-CoV.

## Methods

### Ethics statement

The study of animals was approved by the Institutional Animal Care and Use Committee at the New York Blood Center (Approval #194.14). All animal studies were carried out in strict accordance with the recommendations of the American Veterinary Medical Association (AVMA) Guidelines and the approved protocols.

### Cell lines

Huh-7, HEP-G2, HT-1080, MT-2, Hep-2, Caco-2, HeLa, 293T, ACE2/293T, A549, NBL-7, PK15, MDCK, FRhK-4, Vero, Vero E6, and MA-104 cells were used for detection of pseudovirus infectivity and for identification of expression of MERS-CoV’s receptor DPP4. Information on these cell lines as well as their origins and providers was specifically described in Table 1.

### Recombinant plasmid construction

The codon-optimized genes expressing full-length S protein of MERS-CoV (humanbetacoronavirus 2c EMC/2012, hCoV-EMC, GenBank accession no. AFS88936.1) were synthesized by GenScript (Nanjing, China) by replacement of the N-terminal signal peptide (residues 1–17) with CD5 signal sequence and insertion into pcDNA3.1 vector (Invitrogen, Carlsbad, CA). The constructed recombinant MERS-CoV plasmid (rMERS-CoV-S) containing the S gene was confirmed for correct insertion by sequencing analysis.

### Production of MERS-CoV pseudovirus bearing S protein

Generation of MERS-CoV pseudovirus was done as previously described with some modifications [10]. Briefly, 293T cells (ATCC, Manassas, VA) were co-transfected with 20 μg of plasmid encoding Env-defective, luciferase-expressing HIV-1 (pNL4-3.luc.RE) and 20 μg of rMERS-CoV-S plasmid, respectively, into a-T175 tissue culture flask using the calcium phosphate method. Cells were changed into fresh DMEM 8 h later. Supernatants were harvested 72 h post-transfection and used for single-cycle infection. The plasmids encoding vesicular stomatitis virus G protein (VSV-G-pcDNA3.1) and pcDNA3.1 vector were co-transfected with pNL4-3.luc.RE plasmid to generate VSV-G pseudovirus and pseudovirus without Env (Env-) as controls.

### HIV-1 p24

The p24 content in the produced MERS-CoV pseudovirus was quantified by ELISA, as previously described, with some modifications [10]. Briefly, ELISA plates were precoated with HIVIG (5 μg/ml) overnight at 4°C and blocked at 37°C for 2 h. Lysed pseudovirus was added to the plates and incubated at 37°C for 1 h. After washes, the plates were respectively incubated with anti-p24 mAb (183-H12-5C, 1:20) and then with horseradish peroxidase (HRP)-conjugated goat anti-mouse IgG at 37°C for 1 h. The substrate 3,30,5,50-tetramethylbenzidine (TMB) (Zymed, Carlsbad, CA) was added, and the reaction was stopped by 1 N H_2_SO_4_. The absorbance at 450 nm (A450) was measured by ELISA Plate Reader (Tecan, San Jose, CA).

### Detection of MERS-CoV pseudovirus infectivity

To detect infectivity, different target cells (10^4^/well in 96-well plates) from various sources (Table 1) were respectively infected with MERS-CoV pseudovirus. Fresh DMEM was added 12 h post-infection, and RLU was measured 72 h later by Ultra 384 luminometer (Tecan).

### Western blot

Western blot was performed to detect HIV-1 p24 and MERS-CoV S protein in the generated pseudovirus, and to identify expression of DPP4 in cells susceptible to MERS-CoV pseudovirus as previously described with some modifications [10]. Briefly, lysed pseudovirus (200 ng/ml p24) or cell lysates (30 μg total proteins) were respectively separated by 10% Tris-glycine gels, which were then transferred to nitrocellulose membranes. After blocking with 5% non-fat milk in PBST overnight at 4°C, the blots were respectively incubated with anti-p24 (183-H12-5C, 1:50), anti-S of MERS-CoV (1:200) (prepared in our laboratory), and goat anti-DPP4 (1 μg/ml, R&D Systems, Minneapolis, MN), for 1 h at room temperature. After three washes, the blots were then incubated with HRP-conjugated anti-mouse IgG (for HIV-1 p24 and MERS-CoV S, 1:3,000, Invitrogen) or anti-goat IgG (for DPP4, 1 1:3,000, R&D Systems) for 1 h at room temperature. Signals were visualized with ECL Western blot substrate reagents and Amersham Hyperfilm (GE Healthcare).

### Expression and purification of recombinant protein

The construction, expression and purification of the recombinant protein fused with Fc (S-RBD-Fc) were done as previously described [11,25]. Briefly, genes encoding RBD (residues 377–662) of MERS-CoV S protein were amplified by PCR using synthesized codon-optimized full-length S sequences of MERS-CoV as the template, which were then digested by EcoR I and Bgl II restriction enzymes and inserted into the pFUSE-hIgG1-Fc2 expression vector (hereinafter named Fc, InvivoGen, San Diego, CA). The sequence-confirmed recombinant plasmid was transfected into 293T cells (ATCC, Manassas, VA) seeded 24 h before transfection, followed by replacement of culture medium by serum-free DMEM (Invitrogen, Carlsbad, CA) 10 h later and then collection of supernatant containing expressed protein 72 h post-transfection. The recombinant protein was purified by Protein A affinity chromatography (GE Healthcare, Piscataway, NJ), according to the manufacturer’s instructions.

### Mouse vaccination and serum collection

Mice were subcutaneously immunized with 10 μg/mouse of recombinant MERS-CoV S-RBD-Fc protein formulated with Freund’s complete adjuvant (Sigma, St. Louis, MO) and boosted once with 5 μg/mouse of the immunogen and Freund’s incomplete adjuvant at 2-week intervals. Sera collected at 10 days post-2^nd^ vaccination were used to detect neutralizing activity.

### MERS-CoV pseudovirus inhibition assay

Pseudovirus inhibition assay was established to detect neutralizing activity of vaccinated mouse sera and inhibitory ability of antiviral agents against infection of MERS-CoV pseudovirus in target cells [10]. Briefly, pseudovirus-containing supernatants were respectively incubated with serially diluted mouse sera or synthetic compounds at 37°C for 1 h before adding to target cells preplated in 96-well culture plates (10^4^ cells/well). Twenty-four hours later, cells were refed with fresh medium, which was followed by lysing cells 72 h later using cell lysis buffer (Promega) and transferring the lysates into 96-well luminometer plates. Luciferase substrate (Promega) was added to the plates, and relative luciferase activity was determined. The inhibition of MERS-CoV pseudovirus was presented as % inhibition.

### MERS-CoV inhibition assay

Neutralizing antibody titers of mouse sera against infection by MERS-CoV live virus were further detected as previously described for SARS-CoV with some modifications [26,27]. Briefly, serial dilutions of mouse sera were incubated with 100 50% tissue culture infective doses (TCID_50_) of virus for 1 h at 37°C prior to addition to the monolayer of Vero E6 cells in triplicate. Virus supernatant was removed and replaced with fresh medium after 1 h of culture at 37°C. Cytopathic effect (CPE) in each well was observed daily and recorded on day 3 post-infection. The neutralizing titers of mouse antisera that completely prevented CPE in 50% of the wells (NT_50_) were calculated as before [26-28].

### Cytotoxicity assay

The *in vitro* cytotoxicity of the synthetic compounds to Huh-7 target cells was measured by the XTT assay as previously described with some modifications [29]. Briefly, 100 μl of serially diluted compounds in non-color 1640 medium were added to equal volumes of cells (5 × 10^5^/ml) in 96-well tissue culture plates. After incubation at 37°C for 3 days, 50 μl of XTT solution (1 mg/ml) containing 0.02 μM of phenazinemethosulphate (PMS) were added. Four hour later, the absorbance at 450 nm (*A*450) was measured with ELISA Plate Reader.

## Competing interests

The authors declare that they have no competing interests.

## Authors’ contributions

LD, YZ and SJ designed the research. GZ, LD, CM, YL, LL, VKP, LW, FY, and BJZ performed the research. GZ, LD, and YZ analyzed the data. LD, YZ, and SJ wrote and modified the paper. All authors read and approved the final manuscript.
